# Design of polarization-insensitive high-visibility silicon-on-insulator quantum interferometer

**DOI:** 10.1038/s41598-018-32769-5

**Published:** 2018-10-02

**Authors:** Jingjing Zhang, Kai Guo, Minghong Gao, Yang Gao, Junbo Yang

**Affiliations:** 1 Center of Material Science, College of Liberal Arts and Sciences, National University of Defense Technology, Changsha, 410073 China; 2 College of Advanced Interdisciplinary Studies, National University of Defense Technology, Changsha, 410073 China; 3 College of Artificial Intelligence, National University of Defense Technology, Changsha, 410073 China; 4 Xi’an Research Institute of High-Technology, Xi’an, 710025 China

## Abstract

We based on integrated silicon-on-insulator platforms design the key components of an on-chip interferometer, beam splitter and directional coupler included, valid in high-visibility interference at telecommunication wavelengths. Special attention is given to the equal-proportion beam splitting and directional coupling, which is achieved by carefully designing the geometric dimension of multi-mode interferometer structure. The proposed interferometer facilitates low loss, broad operating bandwidth, anticipated large tolerance on size variation induced in fabrication procedures, based on a particular wafer with silicon layer thickness of 320 nm. The most highlight property of polarization-insensitive, enables the path-selective qubits generation for bi-polarization that further makes possible quantum key distribution using high dimensional protocols. We numerically demonstrate interference at 1550 nm with visibilities of 99.50% and 93.99% for transverse-electric and transverse-magnetic polarization, respectively, revealing that the proposed interferometer structure is well capable of on-chip optical control especially in quantum optics regime.

## Introduction

Quantum key distribution (QKD) is arguably the most mature technology in quantum optics^1^, which facilitates high security communication using optical cryptography in the single-photon regime. Behaving in a probabilistic manner, the random characteristics of photons are applied to suffice various quantum protocols, such as E91^2^ and BB84^3^ where qubits can be encoded in polarization. More specifically, efficient quantum interference is the key resource of both the discrete-variable protocols using entangled photons^4^, and the discrete-modulated protocols using weak coherent pulses^5^. While the initial quantum interference experiments were carried out by employing free-space devices^6,7^, the results came across huge challenge to build up robust system. On the other hand, although the quantum interferometer set ups using fiber-based devices take the merits of low loss, compact coiling and easy fabrication^8,9^, there remains a challenge to mitigate huge polarization variation and dramatic bending loss when small footprint of the investigated system is desired.

Silicon-on-insulator (SOI) platforms attract great research interest in the past decade due to their high compatibility with the Complementary Metal-Oxide-Semiconductor (CMOS) technology and mature fabrication procedures^10^. Being significant building blocks of on-chip optical communication systems, a variety of electric- or optical-driven components, including beam splitters^11^, polarization splitters^12^, wavelength division multiplexers^13^, band-pass filters^14^ *et al*., can be compactly integrated within a single chip. Moreover, silicon waveguide enables nonlinear coefficient of several orders of magnitude higher than highly nonlinear fibers, in addition to have easy-filtered narrow-linewidth Raman scattering response^15,16^. Thus, it becomes appealing as SOI components can facilitate correlated photon-pair generation via spontaneous four-wave mixing^17^ and quantum interference simultaneously^18^.

**Figure 1:**
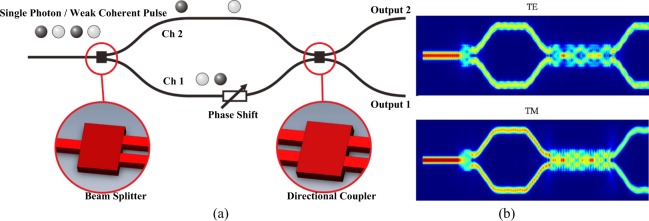
(**a**) The schematic of the proposed interferometer structure, and (**b**) energy distribution for both polarizations.

However, stricter requirements of the main designable components in on-chip interferometer, beam splitters and directional couplers, become emerged in quantum optics regime than that in classical optics regime. Few of previous studies^11–14,19–21^, to our best knowledge, notice that the probable visibility reduction caused by unequal-proportion directional coupling, may be acceptable for classical applications, but significantly lower the accuracy of quantum cryptography^22^. Moreover, it is appealing if quantum interferometer operates polarization-independently, that is, efficient interference can be achieved for bi-polarization, which further enables high-dimensional QKD involving polarization state and path selection as two independent protocols. Hence, we propose a Mach-Zehnder interferometer structure, which consists of an incident beam splitter with one input and two outputs, two propagation waveguides where the heat-induced or electric-driven phase shift can be added, and a directional coupler with two inputs and outputs. The air-cladded interferometer originally comes from a SOI wafer, with a silicon layer (thickness determines the height of wavaguide cross-section) on top of a silica box layer (thickness of 3 *μ*m in simulation). All of the single-mode waveguides have a cross-sectional width of 500 nm. Note that the incident beam splitter can be sometimes replaced by directional coupler with one input being setted idly. This issue, however, comes across the risk that cross-coupling may take place between two input waveguides of directional coupler that brings uncertainty of beam incidence. In addition, the proposed interferometer remains valid by assuming that only one trail of single photon or weak coherent pulse is incident and probabilistically goes along channel 1 or channel 2, as shown in Fig. 1(a).

The key resource of both beam splitter and directional coupler is the design of the multi-mode interferometer (MMI) structure, which takes a simple structure but works well learnt from self-imaging theory^19^. The incident beam polarized in either transverse-electric (TE) mode or transverse magnetic (TM) mode, stimulates high-order modes in MMI that focus on different position. Some field profiles of high-order modes are similar to that stable in single-mode waveguides, which makes possible arbitrarily energy redistribution (see Fig. 1(b)). Moreover, MMI length facilitating efficient focusing often comes from the beat length between fundamental mode and first-order mode given by *L*_*π*_ = *π*/Δ*β*, where Δ*β* = |*β*_0_ − *β*_1_| denotes the inter-mode propagation constant difference. Specifically, MMI length follows *L* = 3*ML*_*π*_/4*N*, where integers $M\geqslant 1$ and *N* denote the focusing order and the focusing points amount, respectively. Note that *M* often equals 1 corresponding to the shortest MMI length and the lowest propagation loss^23^. Additionally, as MMI width and height, under fully-etched fabrication assumption, determine the effective refractive index *n*_eff_ that follows *β* = 2*πn*_eff_/*λ*, it is valid to achieve beam splitting and directional coupling for both polarizations via careful geometric design.

## Results

**Figure 2:**
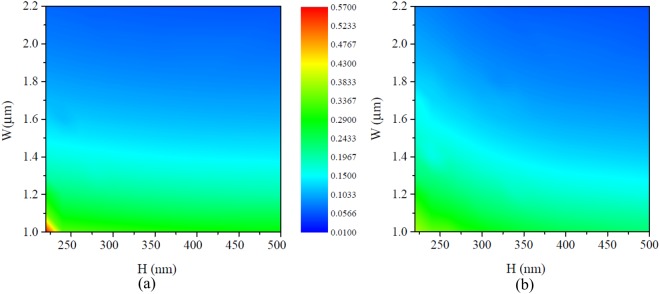
The inter-mode effective refractive index difference versus MMI height and width for (**a**) TE and (**b**) TM polarization.

Special attention of designing the MMI is given to the inter-mode effective refractive index difference between the fundamental modes and the first-order modes, Δ*n*_eff_ = |*n*_eff,0_ − *n*_eff,1_|, where the critical polarization-insensitive structure facilitates the same Δ*n*_eff_ for both polarizations, that is, Δ*n*_eff_(TE) = Δ*n*_eff_(TM). Hence, the beat lengths with respect to two polarizations take the same value, i.e. *L*_*π*_(TE) = *L*_*π*_(TM), which can be achieved by carefully optimizing the cross-sectional dimension (height H and width W) of MMI. The numerical optimization is carried out through the Finite-Difference Time-Domain (FDTD) mode solver^24–26^. Figure 2(a,b) show that Δ*n*_eff_ at 1550 nm reduces with increasing width and height for both polarizations, yet the increasing height impacts more for TM polarization revealing the fact that electric field dominantly distributes along height direction. In particular, when MMI height and width reach 320 nm and 1.58 *μ*m, the effective refractive index for TE_00_, TE_01_, TM_00_, TM_01_ modes reach 3.0361, 2.9160, 2.6271 and 2.5069, respectively, which enables Δ*n*_eff_(TE) = 0.1201 being most approximate to Δ*n*_eff_(TM) = 0.1203. Note that by using an unique height of 320 nm, the resulting polarization-independent beat length and the capable minimal MMI length are only 6.44 *μ*m and 2.4 *μ*m, respectively, while by using conventional height values of 220 nm^27^, 250 nm^28^ and 340 nm^20^, there remains a challenge to trade off Δ*n*_eff_ for both polarizations involving MMI width as the only degree of freedom. As MMI height heavily determined by SOI wafers is often fixed in actual cases, the optimized value of 320 nm is mainly concerned in the following discussions.

The normalized transmittances at the proposed beam splitter outputs (subscripted as Ch1 and Ch2) are used to evaluate the high-efficiency and equal-proportion characteristics of beam splitting functionality. Note that when two propagation waveguides of the proposed interferometer are applied as nonlinear medium for photon-pair generation^18^, high-efficiency and equal-proportion beam splitting ensures enough and balanced pump power to drive spontaneous four-wave mixing. On the other hand, when the proposed interferometer is used for e.g. path-selective QKD, high-efficiency beam splitting reduces the risk of losing photons, meanwhile equal-proportion beam splitting ensures photons to equiprobably go along two paths. Based on the optimized height of 320 nm, the transmittances for both polarizations are simulated involving MMI width, MMI length and gap width (the distance between two single-mode waveguides at MMI input/output) between output waveguides as degrees of freedom.

**Figure 3:**
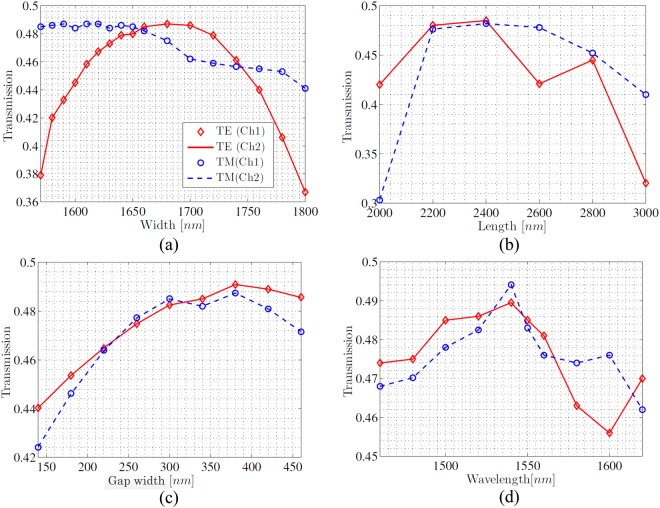
The normalized transmittances of the proposed beam splitter versus (**a**) MMI width (**b**) MMI length (**c**) gap width of two output waveguides (**d**) wavelength. TE polarization and Channel 1: red diamond; TE polarization and Channel 2: red solid; TM polarization and Channel 1: blue circle; TM polarization and Channel 2: blue dashed.

Figure 3(a) shows the transmittances versus MMI width, with MMI length of 2.4 *μ*m and gap width of 320 nm. The MMI widths in-between 1.55 *μ*m and 1.65 *μ*m enable high transmittance exceeding 48% for TM polarization, meanwhile the highest transmittance for TE polarization takes place at a width of 1.68 *μ*m. The electric field dominantly distributes along height direction for TM polarization, thus the variation of MMI width brings a negligible contribution to the resulting transmittance. On the other hand, the electric field dominantly distributes along width direction for TE polarization, thus there exists an optimized width that facilitates the strongest inter-mode coupling, that is, the highest transmittance. For a MMI width of 1.66 *μ*m, the total transmittance by summing up those at two outputs, reach 97% and 96.4% for TE and TM polarization, respectively, which is comparable with previous works^21,29^ but facilitates near-unity equal-proportion beam splitting as the transmittance at output 1 approximately overlaps with that at output 2. Note that by trading off transmittances for both polarizations, such an optimized width slightly differs from the previously presented value. Figure 3(b) shows the transmittances versus MMI length, with MMI width of 1.66 *μ*m and gap width of 320 nm The high transmittance exceeding 48% is achieved when MMI length is in-between 2.2 *μ*m and 2.4 *μ*m. Such an optimized MMI length is approximate to that estimated from the beat length, in addition to indicate a good polarization-insensitive behaviour. Figure 3(c) shows the transmittances versus gap width, with MMI width and length of 1.66 *μ*m and 2.4 *μ*m, respectively. The highest transmittance is obtained when gap width reaches 380 nm, yet the gap width of 320 nm enables the same transmittance of 48.4% for both polarizations. Since the gap width determines the transverse positions of two single-mode waveguides, the highest transmittance is achieved when the fundamental mode profile in two single-mode waveguides matches well with the first-order mode profile in MMI. Additionally, based on the optimized geometric dimension, Fig. 3(d) shows the transmittances versus wavelength, where the highest value takes place at 1540 nm, meanwhile the closet value for both polarizations takes place at 1550 nm. The total transmittances at all concerned wavelengths exceed 90%, demonstrating that the 1 dB bandwidth of the proposed beam splitter includes the *C*, *L* and *S* telecommunication bands ranging from 1460 nm to 1625 nm.

**Figure 4:**
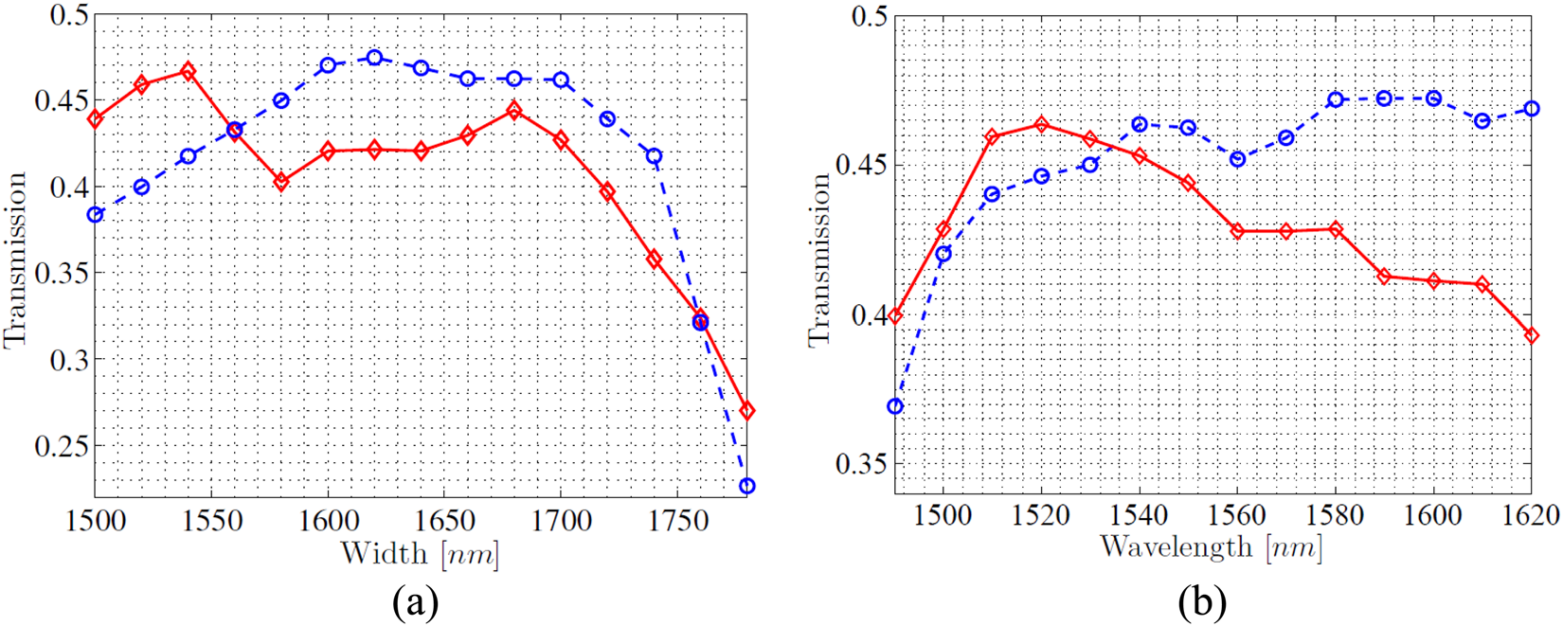
The normalized transmittances of the proposed directional coupler versus (**a**) MMI width (**b**) wavelength. TE polarization and Channel 1: red diamond; TE polarization and Channel 2: red solid; TM polarization and Channel 1: blue circle; TM polarization and Channel 2: blue dashed.

The two propagation waveguides of the proposed interferometer need to have same cross-sectional dimension, which can be designed particularly to suffice various requirements, for example, near-zero anomalous group-velocity dispersion for photon pair generation^30^. However, only approximate propagation loss for two polarizations is concerned in this work, where the cross-sectional dimension of 320 × 500 nm results in simulated loss coefficient of 1.60 dB/cm. Moreover, gap width of 320 nm is followed, meanwhile MMI width and length are recalculated in designing directional coupler. Figure 4(a) shows the transmittances versus (directional coupler) MMI width (of directional coupler), length of 4.8 *μ*m that enables two-to-two focusing^19^. The equal-proportion beam coupling remains unchanged, meanwhile the optimized width of 1.68 *μ*m enables high transmittance of 44.4% and 46.23% for TE and TM polarization, respectively. Figure 4(b) shows that transmittance trends smaller and larger with increasing wavelength, and at 1540 nm takes approximate values of 45.3% and 46.35% for TE and TM polarization. In the spectral range in-between 1460 nm and 1625 nm, the proposed directional coupler achieves high total transmittance exceeding 70%.

**Figure 5:**
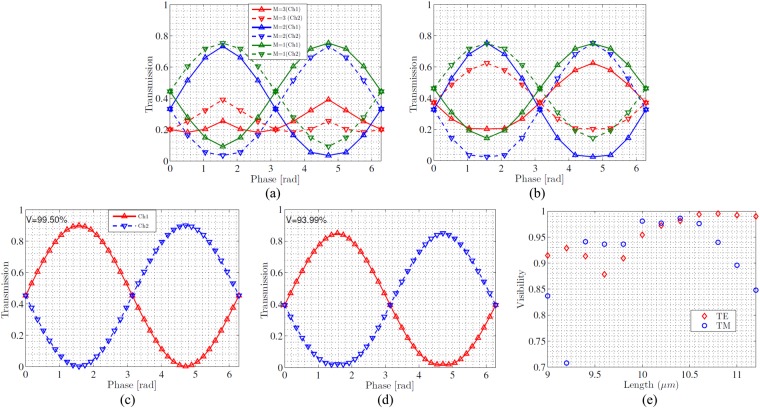
The normalized transmittance versus phase shift added in Channel 1 for (**a**) TE polarization and (**b**) TM polarization. The interference simulation for (**c**) TE polarization and (**d**) TM polarization when MMI length reaches 10.8 *μ*m. (**e**) The calculated visibility versus (directional coupler) MMI length.

The MMI length heavily determines transmittance and beam splitting ratio of directional coupler, which further impacts the interference visibility using proposed interferometer. To testify this, we compare three MMI lengths, 4.8 *μ*m, 9.6 *μ*m and 14.4 *μ*m, which correspond to focusing orders of *M* = 1, *M* = 2 and *M* = 3, respectively. We use continuous-wave pump incident from beam splitter and introduce a phase shift in channel 1, where transmittance describes the probability of detecting single photon or weak coherent pulse at output 1 and output 2 in Fig. 1(a). Figure 5(a,b) show transmittance versus phase shift for TE and TM polarization, respectively, where the maximum takes place at *M* = 1, meanwhile the minimum takes place at *M* = 2. The resulting visibility reach 78.28%, 90.95% and 36.38% for TE polarization, and 67.74%, 93.64% and 40.31% for TM polarization, when *M* is 1, 2 and 3, receptively. The MMI length corresponding to *M* = 1 comes across the risk of incomplete inter-mode coupling between fundamental modes and first-order modes, meanwhile the MMI length corresponding to *M* = 3 suffers from huge propagation loss with respect to first-order modes. By trading off efficient inter-mode coupling and low propagation loss, the optimized focusing order is *M* = 2. Moreover, when MMI length reaches 10.8 *μ*m, the proposed interferometer achieves visibilities of 99.5% and 93.99% for TE and TM polarization, respectively (see Fig. 5(c,d)). A higher visibility of 98.63% for TM polarization can be achieved at a MMI length of 10.4 *μ*m, yet the visibility for TE polarization reduces down to 98.18%. In addition, the visibility for both polarizations remains exceeding 95% with MMI lengths in-between 10 *μ*m and 10.6 *μ*m, demonstrating that the proposed interference has a large tolerance on longitudinal size variation of directional coupler which may be induced in fabrication procedures (see see Fig. 5(e)).

While a number of MMI-based directional coupler designs have been carried out in previous studies, few of them gives attention to suffice high-visibility interference, especially taking both polarizations into account. By using numerical data from several representable studies, visibilities of 96.26%^31^ and 98.8%^32^ are calculated for TE polarization, yet the interferometer using these directional coupler structures fail to realize interference for TM polarization. Furthermore, this work, where high-visibility quantum interference can be anticipated, may make higher previous experimental visibility of only 80.2%^33^.

## Discussion

We based on integrated silicon-on-insulator platforms propose an on-chip interferometer, which becomes a promising design being capable of quantum interference in the single-photon regime. By carefully designing the geometric dimension of multi-mode interferences used in beam splitter and directional coupler, the proposed interferometer facilitates low loss within a broad bandwidth covering telecommunication spectral range, in addition to achieve large tolerance on size variation enabling reliable and reproducible performance. We present that, by using the silicon-on-insulator wafer with silicon layer thickness of 320 nm, these merits are valid for both polarizations, where a polarization-independent path-selective quantum key distribution can be anticipated in future experimental investigations. We numerically demonstrate that at 1550 nm, visibilities of 99.50% and 93.99% for transverse-electric and transverse-magnetic polarization, respectively, can be achieved. To sum up, the proposed interferometer with advantages of simply-designed, easily-fabricated, and bi-polarization-available, holds great potential in efficient optical control especially in quantum optics regime.

## Methods

### Interference simulations and visibility calculation

**Figure 6:**
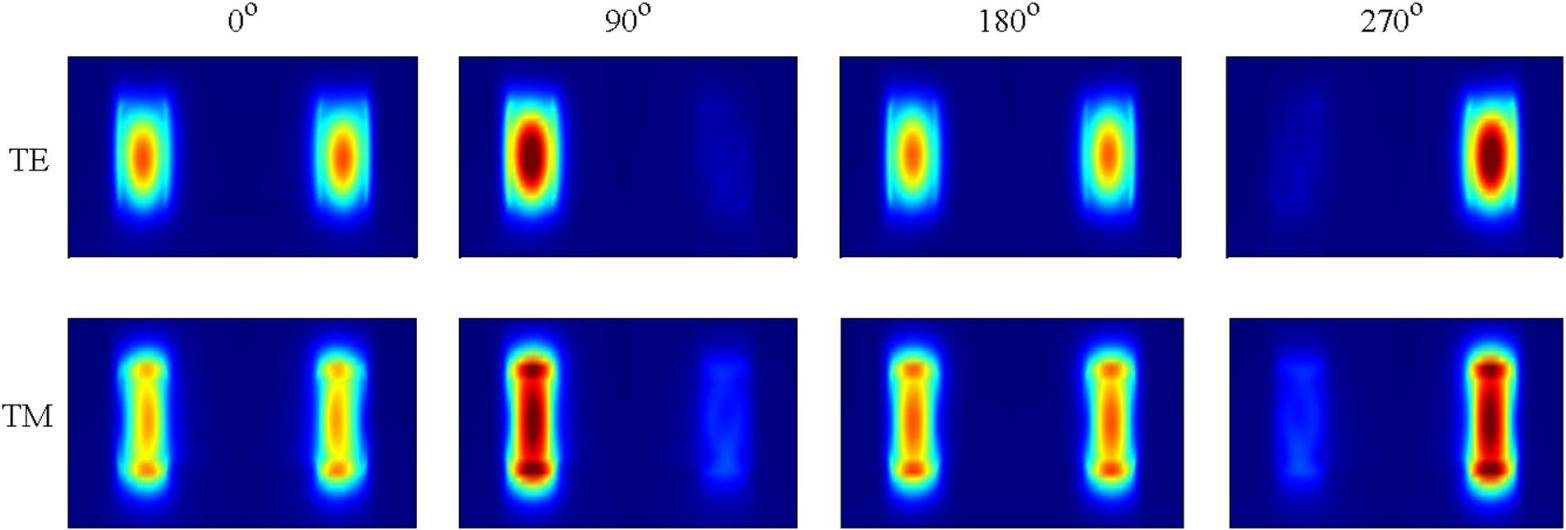
The output mode profile for TE and TM polarizations with respect to different phase shift.

Based on the proposed interferometer, we via FDTD mode solver simulate interference process and calculate the classical visibility that can be equivalenced to the probability study in quantum interference. A continuous-wave beam at 1550 nm incident in input of beam splitter and is equally separated into two arms, one of which phase shift is added. The beam power coupled out of two outputs of directional coupler is counted (see Fig. 6) with respect to phase shift. Hence, the classical visibility is calculated through

1 $$V=\frac{{P}_{{\rm{\max }}}-{P}_{{\rm{\min }}}}{{P}_{{\rm{\max }}}+{P}_{{\rm{\min }}}}$$

where *P*_max_ and *P*_min_ denote the maximal and minimal power at output 1 (or output 2).

## Data Availability

The datasets generated and analyzed during the current study are available from the corresponding author on reasonable request.
